# Economics of Audiology Wages: Benchmarking a Doctoral Health Profession Within U.S. Healthcare

**DOI:** 10.1055/s-0046-1825788

**Published:** 2026-10-06

**Authors:** Victor Bray, Amyn M. Amlani

**Affiliations:** 1 Osborne Audiology Department, College of Nursing and Health Professions, Drexel University, Elkins Park, Pennsylvania, United States; 2 Department of Communication Sciences and Disorders, College of Allied Health, University of Oklahoma Health Sciences Center, Oklahoma City, Oklahoma, United States

**Keywords:** Audiology wages, Credential-wage decoupling, Healthcare workforce economics, Reimbursement policy

## Abstract

Audiology requires doctoral-level education, yet its earnings trajectory has remained comparatively modest within U.S. healthcare. This article examines historical trends in audiology wages using national Bureau of Labor Statistics (BLS) Occupational Employment and Wage Statistics (OEWS) data, benchmarking absolute wage levels, growth trajectories, and degree-based comparisons against 33 other healthcare occupations. Results indicate that the rate of wage growth slowed following the profession's transition to doctoral-level entry relative to the preceding master's-dominant period. Audiology wages also remained closely coupled to speech-language pathology wages throughout the study period. When benchmarked against healthcare professions grouped by minimum educational requirement, audiology's mean wage clustered within the master's-degree wage band rather than the doctoral-degree band, a pattern consistent with credential–wage decoupling. These findings suggest that increased educational requirements alone were insufficient to reposition audiology's economic standing, with structural factors–including reimbursement dependency, device-anchored revenue models, employment-based compensation structures, and scope fragmentation–implicated as consistent explanations. Earnings function here as a workforce sustainability signal with implications for recruitment, retention, and professional positioning within U.S. healthcare.

## Introduction

The long-term sustainability of a healthcare workforce depends not only on the supply of trained professionals but on whether earnings—encompassing wages, salary, commissions, bonuses, and self-employment income—appropriately reflect educational investment, clinical responsibility, and economic value within the healthcare system. Sustainability is reflected in earnings patterns over time, particularly when compared against occupations with similar training requirements and clinical responsibility. When earnings fail to scale with educational investment and scope, workforce consequences often follow, including recruitment challenges, early-career attrition, and constrained professional mobility. From an economic perspective, earnings function as a revealed signal of how markets, payers, and institutions value professional services in practice.

Audiology represents a unique case within the U.S. healthcare labor market. The profession requires doctoral-level education and extensive clinical training, yet its long-run income trajectory has remained comparatively modest. While audiology compensation has increased since formal recognition by the U.S. Bureau of Labor Statistics (BLS) as a standalone occupation in 1999, 1 the rate of growth and relative positioning within healthcare raise important questions about professional status and economic authority.

This article examines historical trends in U.S. audiology wages and benchmarks those trends against comparable healthcare professions. Consistent with standard workforce analyses, wages are used as the primary empirical measure of earnings, as defined and detailed in the Methods section. Using national wage data from the BLS, we evaluate absolute wage levels, growth trajectories, and degree-based comparisons spanning two decades. These data are interpreted using a descriptive economic framework to assess what wage patterns reveal about audiology's current position within the healthcare system, including human capital return, credential-wage decoupling, and pricing power. This analysis is intentionally bounded: it describes and contextualizes observed wage patterns but does not establish causal mechanisms, attribute outcomes to specific structural factors, or evaluate proposed reforms. The structural conditions consistent with these patterns are identified in the Discussion as interpretive context; their institutional mechanisms and policy implications are the subject of companion analyses in this special issue.

## Methods

In this manuscript, the term “wages” refers, specifically, to annualized employment compensation as reported by the BLS Occupational Employment and Wage Statistics (OEWS). 2 These values represent pay received from employment and do not include bonuses, commissions, or self-employment income.

The term “earnings” is used more broadly to describe total professional income, inclusive of wages, salary, commissions, bonuses, and self-employment income, as reported in U.S. Census data and professional association surveys. 3 When analyses rely exclusively on BLS OEWS data, the term “wages” is used explicitly. 4 When comparing across multiple data sources or discussing professional income more generally, the term “earnings” is used as a conceptual construct.

### Conceptual Framework: Earnings as a Labor-Market Signal of Professional Position

In labor economics, earnings (i.e., professional income) are a central indicator of how occupations are valued within a market. For healthcare professions in particular, earnings reflect the interaction of multiple structural factors, including human capital investment, scope of practice, reimbursement authority, substitutability of services, and the ability to generate and retain revenue independently. Earnings—unlike professional titles, statutory language, or aspirational role definitions—represent a revealed outcome of how employers, payers, and healthcare systems allocate economic value in practice. In applied workforce analyses, “wages” are commonly used as an observable and conservative proxy for earnings, as they reflect employment-based compensation captured in national datasets. 4

A foundational principle of labor economics is the “human capital model,” which predicts that greater investments in education and training should, on average, yield higher lifetime earnings. 5 In healthcare, this relationship is typically reinforced by regulatory structures that grant expanded clinical authority, independent billing privileges, and participation in higher-value care pathways to professions with advanced training. When earnings fail to scale with educational investment, the implication is not diminished skill or social relevance, but a structural disconnect between training and market valuation.

Earnings also convey information about “relative professional position” within the healthcare labor market. 6 When benchmarked against comparable occupations, earnings rankings and long-term growth trajectories signal where a profession sits in the broader hierarchy of healthcare delivery. Professions that occupy central, autonomous roles—particularly those with direct reimbursement authority and control over service pricing—tend to cluster toward the upper end of earnings distributions within degree cohorts. Conversely, professions whose services are mediated through other providers or tied to commoditized products often exhibit lower relative earnings and more compressed income distributions, even when training requirements are substantial.

Another relevant economic concept is “earnings compression,” defined as relatively narrow dispersion of income across experience levels or percentile ranks within a profession. 6 In applied labor-market analysis, compression often signals constrained pricing authority, limited-service differentiation, or compensation structures that cap revenue generation regardless of expertise or tenure. In healthcare settings, such compression typically reflects structural limits on independent service valuation, resulting in muted upward income mobility even as training requirements and clinical responsibilities expand. A related but distinct pattern is credential-wage decoupling, in which a profession's market wage position falls below the tier predicted by its educational requirements—reflecting structural constraints on how advanced training is recognized and valued within healthcare payment systems.

Earnings growth over time provides additional insight into professional positioning. 7 Longitudinal compensation trends can indicate whether a profession's economic role is strengthening, stagnating, or eroding relative to peers. When analyzed across extended periods, growth slopes may reveal structural shifts associated with changes in education requirements, regulatory environments, or healthcare delivery models.

Wage trends in this analysis are reported in nominal dollars for two reasons. First, nominal values reflect labor-market valuation as experienced by workers and employers at the time—the signal that shapes hiring, enrollment, and retention decisions. Second, because all occupations in this analysis are compared within the same calendar years, inflation affects each series equally and does not alter relative growth slope comparisons across professions. Inflation adjustment is therefore unnecessary for the benchmarking questions addressed here.

It is worth noting that when expressed in real (inflation-adjusted) terms, audiology's wage growth rate over the study period is further attenuated. Between 1999 and 2024, cumulative CPI inflation exceeded 90%, meaning nominal gains that appear modest in absolute terms represent even more limited growth in purchasing power. This consideration reinforces rather than contradicts the directional findings reported here.

In the context of healthcare workforce analysis, earnings, therefore, serve as a composite signal integrating education, authority, and market power. 8 Interpreting earnings trends through this framework allows for a descriptive assessment of professional status without reliance on aspirational goals or proposed reforms. This approach is particularly valuable for understanding how a profession is currently positioned within the healthcare system and how that positioning may influence workforce stability, recruitment, and long-term sustainability.

## Results

### Wage Growth Over Time

Longitudinal trend analysis was used to assess whether audiology wages increased steadily in nominal terms across two periods: the master's degree–dominant period (1999–2006) and the doctoral degree–dominant period (2007–2024), as shown in Fig. 1 . While doctoral entry into practice was formally implemented in 2007, the transition in workforce composition occurred gradually over subsequent years as master's-trained clinicians continued to represent a substantial proportion of the practicing workforce. Linear regression analysis was applied with calendar year specified as the independent variable ( *x* ) and mean audiology wages as the dependent variable ( *y* ). The slope of each regression line represents the estimated annual change in wages (U.S. dollars per year).

**Fig. 1:**
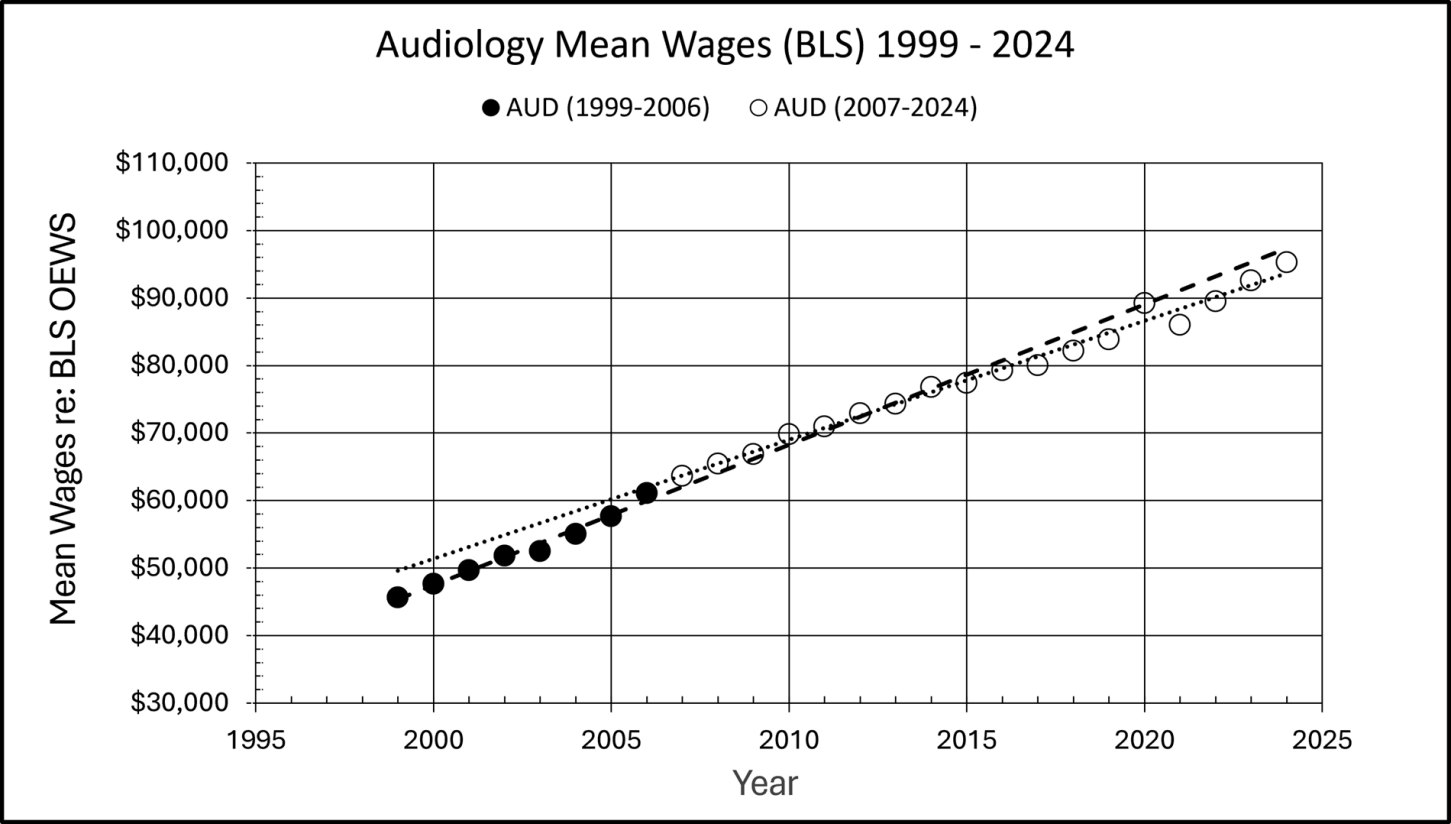
Longitudinal audiology wages, with regression slopes, reported for each period during the master's-dominant (filled circles, dashed line) and doctoral-dominant (open circles, dotted line) preparation periods, with linear regression slopes reported for each period.

Comparison of regression slopes—using the data shown in Fig. 1 —indicates that the rate of wage growth slowed following the transition to doctoral-level education. Specifically, during the master's-dominant period (pre-2007), mean wages increased at a higher annual rate (slope = $2,080) than during the subsequent AuD-dominant period (slope = $1,764). Despite increased educational requirements, the post-2007 AuD entry-level degree period does not exhibit accelerated wage growth. From an economics perspective, this pattern suggests that the transition to doctoral-level preparation was not associated with an increase in the marginal rate of wage growth.

### Absolute Wage Levels

Longitudinal comparison of audiology and speech-language pathology wages reveals closely coupled trajectories over the 1999 to 2024 period ( Fig. 2 ). Annual differences between mean wages for the two professions are small (i.e., $256 greater favoring audiology), frequently change sign, and rarely exceed ± $2,000. Notably, this absence of sustained wage divergence persists across audiology's transition from master's-level preparation (1999–2006) to doctoral-level entry (i.e., $91 greater favoring audiology), indicating that the healthcare labor market continues to price audiology and speech-language pathology similarly despite divergence in degree requirements. This pattern suggests the market has not repriced audiology for doctoral-level preparation relative to a master's-level peer profession.

**Fig. 2:**
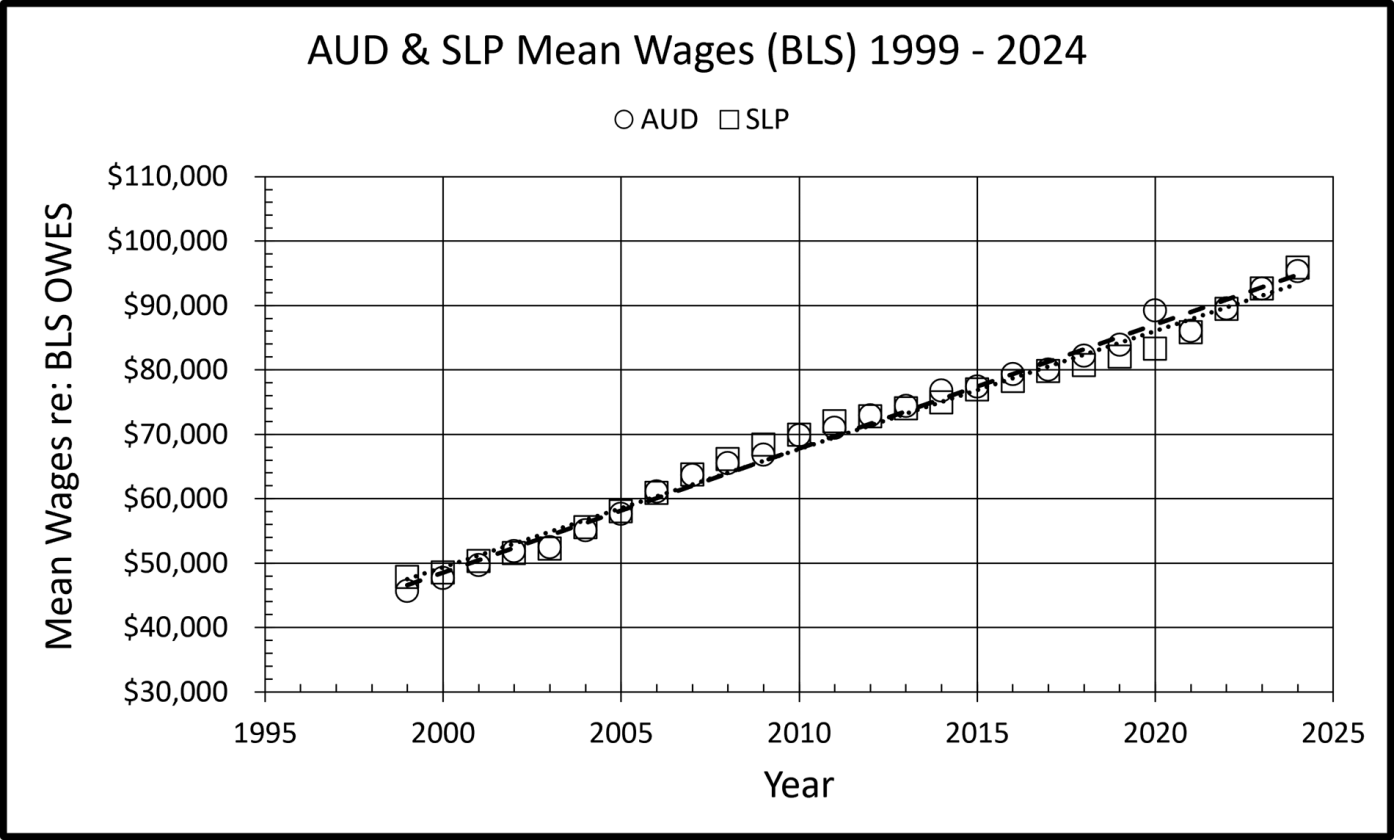
Comparison of mean annual wages for audiology (open circles, dashed line) and speech-language pathology (open squares, dotted line) from 1999 to 2024 using U.S. Bureau of Labor Statistics data.

### Degree-Based Benchmarking

To contextualize audiology wages within the broader healthcare labor market, healthcare occupations were grouped by minimum educational requirement using classifications from the BLS “Occupational Outlook Handbook.” 2 A total of 34 healthcare occupations were included: 10 associate's-degree occupations, 6 bachelor's-degree occupations, 9 master's-degree occupations, and 9 professional or doctoral-degree occupations ( Appendix A ). Occupations requiring less than an associate's degree or those not directly comparable to clinical healthcare practice were excluded.

**Appendix A:** BLS occupations listed by degree requirement and mean annual wages (2022–2024) for the healthcare professions shown in Fig. 3

Healthcare professions by degree	BLS SOC 2	Mean annual wage (2022–2024)
Associate's degree		
Radiation therapists	29–1124	$104,527
Nuclear medicine technologists	29–2033	$94,793
Dental hygienists	29–1292	$89,547
Diagnostic medical sonographers	29–2032	$88,660
Magnetic resonance imaging technologists	29–2035	$86,383
Respiratory therapists	29–1126	$79,637
Radiologic technologists and technicians	29–2034	$75,083
Health information technology and medical registrars	29–9021	$69,650
Cardiovascular technologists and technicians	29–2031	$69,550
Veterinary technologists and technicians	29–2056	$43,697
Bachelor's degree		
Medical dosimetrists	29–2036	$134,117
Registered nurses	29–1141	$93,973
Dietitians and nutritionists	29–1031	$71,800
Clinical laboratory technologists and technicians	29–2010	$62,440
Recreational therapists	29–1125	$60,857
Exercise physiologists	29–1128	$57,497
Master's degree		
Nurse anesthetists	29–1151	$217,223
Physician assistants	29–1071	$130,887
Nurse practitioners	29–1171	$128,390
Nurse midwives	29–1161	$127,377
Genetic counselors	29–9092	$98,047
Occupational therapists	29–1122	$95,943
Speech-language pathologists	29–1127	$92,643
Orthotists and prosthetists	29–2091	$80,023
Athletic trainers	29–9091	$61,073
Doctoral or professional degree		
Family medicine physicians	29–1215	$240,693
Dentists, general	29–1021	$186,713
Podiatrists	29–1081	$161,483
Optometrists	29–1041	$139,013
Veterinarians	29–1131	$135,227
Pharmacists	29–1051	$133,803
Physical therapists	29–1123	$100,267
Audiologists	29–1181	$92,477
Chiropractors	29–1011	$88,437
All U.S. occupations	NA	$65,097

For each occupation, annual mean wages for the years 2022 to 2024 were averaged to estimate current wage levels. These average values were plotted by degree category in Fig. 3 . Within each category, multiple data points overlap, reflecting distinct occupations with similar degree requirements. Audiology is denoted by the filled black circle shown in the Doctorate category.

**Fig. 3:**
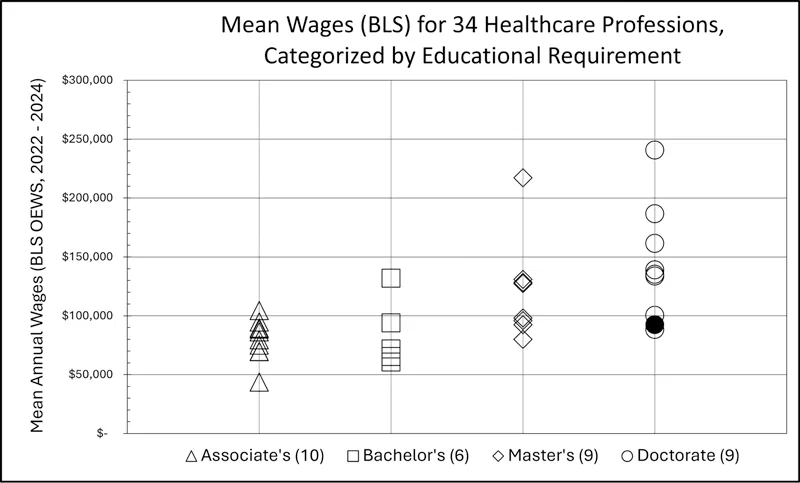
Average annual wages (2022–2024) for 34 healthcare occupations grouped by minimum educational requirement. Audiology is denoted by the filled black circle (see Appendix A for details).

Wage levels are stratified within and across degree categories. Associate's-degree occupations (mean: $80,153, range: $43,697–$104,527) and bachelor's degree occupations (mean: $80,216, range: $57,497–$134,117) demonstrate lower wage levels, whereas Master's-degree occupations occupy a higher wage tier (mean: $114,623, range: $61,073–$217,223), and professional and doctoral-level occupations demonstrate the highest overall wage levels (mean: $142,013, range: $88,437–$240,693). For comparison purposes, the mean 3-year wage (BLS, 2022–2024) across all U.S. occupations was reported as $65,097.

Audiology's 3-year mean annual wage ($92,477) was 42% higher than the U.S. population average and exceeded 8 of 10 associate's-degree occupations and 4 of 6 bachelor's-degree occupations. However, audiology wages exceeded only 2 of 9 master's-degree occupations and 1 of 9 professional or doctoral-level occupations, placing audiology near the lower bound of wages among all 18 higher education-level healthcare professions.

To assess whether observed wage stratification across degree categories reflected systematic separation rather than visual clustering alone, a descriptive nonparametric comparison was performed across associate's-, bachelor's-, master's-, and doctoral-level occupations. A Kruskal–Wallis test indicated differences in wage distributions across degree categories ( *p* < 0.05), consistent with expected educational stratification in healthcare labor markets. This analysis was conducted to confirm wage stratification differences and support descriptive benchmarking rather than formal hypothesis testing.

Despite requiring doctoral-level preparation, audiology wages cluster within the master's-degree wage band—$92,477 versus a master's-tier mean of $114,623—a pattern consistent with credential-wage decoupling, in which the market has not recognized the profession's educational advancement in its wage positioning. This misalignment, combined with structural constraints on pricing authority and service differentiation, also limits upward earnings mobility within the profession relative to doctoral-level peers.

## Discussion

In healthcare labor markets, earnings function as a composite indicator reflecting how education, scope, reimbursement authority, and service differentiation interact in practice. Unlike stated professional roles or statutory definitions, earnings represent revealed valuation by employers, payers, and institutions.

Professions that combine advanced training with independent service valuation and scalable care delivery tend to exhibit higher mean earnings, broader wage dispersion, and steeper long-term growth trajectories. 8 In contrast, professions characterized by constrained pricing authority or limited-service differentiation often display wage compression and credential-wage decoupling despite high levels of training.

When interpreted through this lens, audiology's historical earnings patterns suggest a profession that has achieved substantial educational advancement without commensurate economic repositioning. This analysis is intended to characterize wage positioning rather than to identify causal mechanisms or policy solutions. From an economic perspective, these findings indicate that increased educational requirements alone were insufficient to alter the profession's wage growth trajectory. In the absence of corresponding changes in service taxonomy, scope recognition, pricing authority, and market structure, additional human capital investment did not translate into higher marginal wage returns.

Limited differentiation and inconsistent classification of audiology services within healthcare payment and delivery systems constrain how clinical expertise is recognized and valued, even as training requirements have expanded. As a result, audiology's professional role remains economically mediated through other providers, devices, or institutional structures rather than independently priced as a distinct clinical service domain. These dynamics provide important context for workforce supply patterns documented elsewhere in this special issue, including recruitment challenges, retention pressures, and constrained geographic distribution.

### Structural Drivers of Credential-Wage Decoupling and Earnings Compression

The historical earnings patterns observed in audiology are consistent with several structural features of the profession that, from a labor economics perspective, constrain earnings growth and limit upward wage mobility. These features do not operate independently; rather, they interact to shape how audiology services are valued and compensated within healthcare markets and are examined in depth in companion analyses of audiology taxonomy, reimbursement, and service classification within this special issue.

First, “reimbursement dependency” plays a vital role. Under current Medicare policy, audiology services are classified primarily as diagnostic supplier services rather than as services delivered by limited-license practitioners 9 , restricting independent billing authority and reinforcing referral-dependent care models. From an economic perspective, constrained pricing authority limits a profession's ability to translate clinical complexity and expanded training into independently valued services, contributing to credential-wage decoupling and muted earnings growth.

Second, “device-anchored revenue models” have historically linked professional income to the sale of hearing technology rather than to time, complexity, or clinical specialization 10 . This linkage positions professional revenue within a product-based economic framework where income is driven by transaction volume rather than service differentiation or longitudinal care management, dampening the economic return on professional services and constraining upward income dispersion 11 .

Third, “employment-based compensation structures” common in hospital systems, physician-owned practices, and large organizations tend to cap long-term income potential and weaken alignment between productivity and compensation. Over time, such structures limit the extent to which accumulated experience and specialization translate into differentiated earnings growth.

Finally, “scope fragmentation” across states and payers contributes to inconsistent service valuation, reducing the transferability of advanced skills across markets and weakening the labor market's ability to price audiology services consistently.

Together, these structural features are consistent with the compressed earnings distributions and modest growth trajectories observed in the historical data 12 . The mechanisms through which they interact—and their implications for taxonomy, revenue generation, and labor deployment—are the subject of the companion structural analysis in this issue.

### Workforce Implications

Labor economics predicts that when earnings do not adequately compensate for training duration, opportunity costs, and educational investment, workforce responses follow. 13 These responses often include reduced entry into training programs, increased sensitivity to debt burden, and higher rates of career diversification or attrition.

As an example, the close coupling of audiology and speech-language pathology earnings suggests that the healthcare labor market continues to value the two professions similarly. This pattern implies that doctoral-level preparation in audiology has not translated into differential pricing or earnings separation from a closely related master's-level profession. Rather than indicating equivalence in clinical scope, this finding reflects comparable economic positioning within healthcare institutions and payer structures.

Within audiology, several indicators align with these predictions, including persistent concerns regarding applicant pipelines, elevated debt-to-income ratios among early-career clinicians, and growing interest in alternative professional pathways. While multiple factors influence workforce behavior, earnings dynamics represent a foundational economic signal shaping long-term workforce stability. These findings motivate further examination of how revenue models and taxonomy interact with labor-market outcomes, explored elsewhere in this issue.

## Conclusion

Historical earnings trends indicate that, despite documented workforce supply constraints, audiology's economic position has been shaped largely by structural features governing reimbursement, pricing authority, and care delivery, which have muted expected wage responses to supply–demand pressures. Despite doctoral-level training and expanding clinical complexity, audiology earnings remain positioned near the lower bound of doctoral-level healthcare professions, with limited upward dispersion relative to peers. From an economics perspective, these patterns are consistent with constrained pricing authority, limited-service differentiation, and compensation models that weaken the link between expertise and income.

Importantly, these findings suggest that earnings are not merely a financial outcome but a core workforce signal. When earnings fail to compensate for educational investment and opportunity cost, predictable workforce responses emerge, including recruitment challenges, heightened sensitivity to debt burden, and reduced long-term retention. As such, earnings dynamics play a central role in shaping workforce stability and access to care.

Economic theory further suggests that durable changes in earnings trajectories are unlikely to occur through incremental adjustments alone. Professions that experience sustained earnings growth typically do so when structural alignment exists among reimbursement authority, service valuation, workforce models, and educational pathways. The structural conditions underlying audiology's current earnings positioning, and what alignment across those conditions would require, are examined in the companion analyses of this special issue.

Collectively, these findings position audiology as a profession with high human capital investment but constrained economic authority within the healthcare labor market. Clarifying this present-state positioning is essential for understanding current workforce challenges and for evaluating future initiatives aimed at improving sustainability. Earnings, in this context, function not as a secondary consideration but as a foundational indicator of professional positioning and long-term viability within the U.S. healthcare system.
